# The Image Identification Application with HfO_2_-Based Replaceable 1T1R Neural Networks

**DOI:** 10.3390/nano12071075

**Published:** 2022-03-25

**Authors:** Jinfu Lin, Hongxia Liu, Shulong Wang, Dong Wang, Lei Wu

**Affiliations:** Key Laboratory for Wide Band Gap Semiconductor Materials and Devices of Education, The School of Microelectronics, Xidian University, Xi’an 710071, China; linjinfu@stu.xidian.edu.cn (J.L.); slwang@xidian.edu.cn (S.W.); crotar@163.com (D.W.); wulei12@foxmail.com (L.W.)

**Keywords:** memristor, artificial neural networks, 1T1R

## Abstract

This paper mainly studies the hardware implementation of a fully connected neural network based on the 1T1R (one-transistor-one-resistor) array and its application in handwritten digital image recognition. The 1T1R arrays are prepared by connecting the memristor and nMOSFET in series, and a single-layer and a double-layer fully connected neural network are established. The recognition accuracy of 8 × 8 handwritten digital images reaches 95.19%. By randomly replacing the devices with failed devices, it is found that the stuck-off devices have little effect on the accuracy of the network, but the stuck-on devices will cause a sharp reduction of accuracy. By using the measured conductivity adjustment range and precision data of the memristor, the relationship between the recognition accuracy of the network and the number of hidden neurons is simulated. The simulation results match the experimental results. Compared with the neural network based on the precision of 32-bit floating point, the difference is lower than 1%.

## 1. Introduction

The artificial neural network is constructed by simulating the connection mode of the brain neural network. There are several mainstream algorithms, such as fully connected neural networks, convolutional neural networks, recurrent neural networks, and generative adversarial neural networks [1,2,3,4,5]. Their basic units are artificial neurons, which perform a non-linear operation on the matrix multiplication result of the input and weights, then input the results into the neurons of the next layer network [6,7]. Since synapse weight requires extreme accuracy, if traditional storage devices are employed in storage, each synapse weight needs to occupy multiple storage units. In a large-scale neural network, the weight data will become huge, such as in Google’s Alpha Go, where the weight data reach 100 Mb [8]. Moreover, the transmission process of large-scale weighted data greatly restricts the speed of artificial neural networks [9,10,11]. Tests on system performance show that for neural networks implemented on the Tensor Processing Unit like the Deep Neural Network, 80% to 90% of the execution time is devoted to memory access [9].

Memristors have been shown to be able to emulate synaptic functions by storing the analog synaptic weight and implementing synaptic learning rules [12,13,14,15,16,17]. The network array constructed by using the memristor-simulated synapse can directly realize the multiplication and addition operations of the parallel input analog signal and the weight matrix, which greatly improves the operation speed of the neural network. The memristor is a two-terminal device composed of a conductor/insulator (or semiconductor)/conductor sandwich structure. This simple structure makes it easy to integrate into the high-density 4F^2^ passive crossover array, and can provide the density and connectivity required for hardware implementation of the neuromorphic computing system [18,19,20]. In recent years, a large number of studies have been reported on the construction of artificial neural networks using memristor-simulated synapses [21,22,23,24,25,26]. However, to use a memristor array to implement a synaptic network, a memristor with high nonlinear volt-ampere characteristics [23,27,28] or a highly nonlinear selector in series [5,29,30,31] is required to minimize the non-idealities impact caused by potential current paths during training and inference operations.

However, take into account the level of technology maturity, attempts to implement memristor neural networks have been plagued by device non-uniformity, resistance level instability, and sneak path currents. Especially in integrated circuits, the damage of components will cause irreversible effects on the whole system. Previous studies have been conducted on the impact of different damage of memristor on the whole through simulation [26]. Here, we have established a fully connected neural network system based on a 1T1R array composed of discrete memristors and MOSFETs. MOSFETs as selectors can effectively reduce the leakage current, thus reducing energy consumption and improving performance. The discrete unit facilitates the replacement of abnormal devices, thereby improving the uniformity of the memristor in the array and maximizing the performance of the network. The single-layer and double-layer neural networks are utilized to complete the recognition function of handwritten images through online training. At the same time, the influence of failed devices in the matrix on network performance is further studied. Finally, the influence of the number of hidden layer neurons in the double-layer network on the network performance is studied by simulation.

## 2. Memristor Cross-Bar Array and System Set-Up

**Figure 1:**
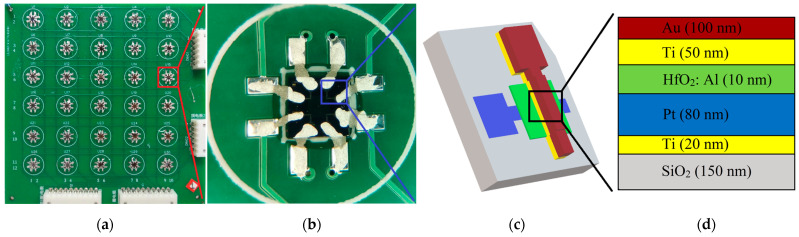
(**a**) The physical diagram of the 1T1R array of 12 rows × 10 columns on a 10 cm × 10 cm PCB. The top electrode of the memristor is connected to the bit line (BL), the bottom electrode is connected to the drain of the NMOS, the source is connected to the source line (SL), and the gate is connected to the word line (WL). (**b**) The micrograph of conductive silver glue lead. (**c**) The three-dimensional schematic diagram of Ti/HfO_2_: Al/Pt RRAM device structure. (**d**) The schematic diagram of RRAM device structure, the thickness of each layer does not represent the true proportion.

In our work, the effects of memristor failure rates and failure modes on network recognition rates is investigated. It is hoped that the unit devices in the matrix can be replaced freely, so the matrix is not integrated on a chip. In order to prevent crosstalk between the memristors in the array, the bottom electrode of each memristor is connected in series with the drain terminal of a commercial NMOSFET (2N7002 type of Jiangsu Changjiang Electronics Technology Co., Ltd., Wuxi, China) to form a 1T1R structure. Among them, the MOS device functions as a selector. Figure S1 demonstrates the excellent performance of MOSFETs. The discrete connection of the array makes it possible to exclude other influencing factors when evaluating the effect of memristor performance. Figure 1a is a distribution diagram of the memristors mounted on the array module, and the corresponding MOS devices are mounted on the back. A 1T1R array of 12 rows × 10 columns is arranged on each 10 cm × 10 cm Printed circuit boards (PCB). There are four discrete memristor devices on each 3 mm × 3 mm bare chip. Connect the memristor electrode pads with the pads on the PCB with conductive silver glue to form a 1T1R matrix structure. Figure 1b is a micrograph of conductive silver glue lead.

The memristor adopts a crossbar structure with an effective area of 5 × 5 µm^2^. The three-dimensional schematic diagram of the Ti/Al: HfO_2_/Pt RRAM device structure is shown in Figure 1c,d, which shows the device stacks structure. The silicon oxide is etched by photolithography to obtain bottom electrode pattern grooves on the silicon oxide substrate. The 20 nm Ti adhesion layer and 80 nm Pt bottom electrode (BE) were evaporated in the groove by electron beam evaporation. The step between the bottom electrode and the substrate is eliminated, and the side wall effect [32] caused by different growth rates in each direction during the deposition of the resistive layer is avoided. Then, the Al-doped HfO_2_ film is deposited by the atomic layer deposition (ALD) method. The 10 nm dielectric layer is deposited alternately by 8 cycles of HfO_2_ and 1 cycle of Al_2_O_3_ at 300 °C. The dielectric layer pattern is obtained by inductively coupled plasma (ICP) etching. Finally, the 50 nm Ti top electrode (TE) and a 100 nm Au cladding layer are deposited by electron beam vapor deposition. Doping HfO_2_ with Al can improve the resistance switching characteristics of the memristor. Figure S2 shows the multi-value storage characteristics and high reliability of the memristor.

**Figure 2:**
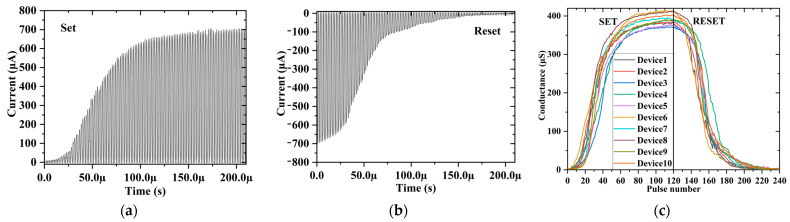
Typical impulse response results of 1T1R unit; 1.8 V/1 μs and −1.8 V/1 μs voltage pulses were used in (**a**) the SET process and (**b**) the RESET process, respectively. (**c**) The conductivity adjustment curve of multiple units under SET/RESET pulse voltage; reading voltage is 0.2 V.

The synaptic weights of the neural network are represented by the resistance of the memristor, which is regulated by voltage pulses. Therefore, the memristor should have the characteristics of multi value storage in pulse mode. As shown in Figure 2a,b, the 1T1R unit is set and reset by a voltage pulse with a width of 1 μs and an amplitude of 1.8 V. Combined with the transient response of the pure HfO_2_ memristor in Figure S3, it can be seen that the conductance of the Al-doped HfO_2_–based memristor can be adjusted gradually by the voltage pulse. Figure 2c compares the conductance changes of 10 devices under pulse excitation. It can be seen that the uniformity of the device conductance changes is acceptable, and they can be adjusted continuously from 1 µS to 350 µS. Compared with integrated circuits, discrete connection has the risk of increasing latency. Figure S4 shows the pulse reading speed of the 1T1R unit, eliminating this risk.

## 3. MNIST Handwritten Digits and Network Training

**Figure 3:**
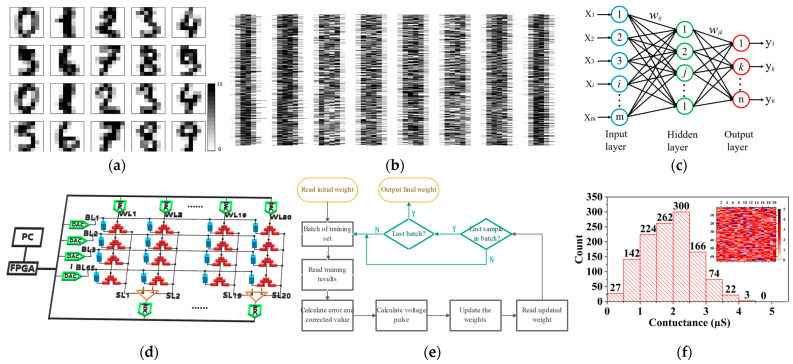
(**a**) Part of the handwritten digits from the MNIST and (**b**) the image storage converted to binary format. (**c**) The schematic diagram of the two-layer fully connected neural network structure. (**d**) The schematic diagram of the single layer fully connected neural network system structure. (**e**) The flow charts of synapse weight training. (**f**) The cumulative distribution of the conductance state of the memristor removing the failed unit; the inset shows the total conductance distribution diagram.

The data set used in this article is the handwritten digits MNIST data set. The training set selected 3823 digital images handwritten by 30 people, and the test set selected 1797 digital images handwritten by 13 people. Each image has 16-level grayscale 8 × 8 pixels, which can be represented by a 4-digit binary number. At the same time, each image has a classification label. Figure 3a,b, respectively, show part of the data set and the image after this part of the data is converted to binary format.

Figure 3c shows the schematic diagram of the two-layer fully connected neural network structure. In our research, we are required to classify image signals of 64-bit pixels in 10 categories. Therefore, the input layer of the neural network requires 65 neurons (64-bit pixel signal plus 1-bit bias signal), and the output layer requires 10 neurons. Since the synapse weight of the neural network has both positive and negative values, the difference between two 1T1R units is used to represent a synapse weight. In general, the size of the memristor array actually needed for a single-layer network is 65 × 20. Figure 3d shows the schematic diagram of the single layer fully connected neural network system structure. The input of the front neuron layer, adaptive synaptic weight, and the output of the posterior neuron layer are consistent with the pulse input from BL, 1T1R units’ conductance, and current output through SL, respectively. 

For a fully connected neural network, the update of the synapse weight can be obtained through the error back propagation algorithm [33]. The training error of a single sample of the neural network is:

(1) $$E(w)=\frac{1}{2}\sum_{k=1}^{n}(z_{k}-y_{k}{)}^{2}$$

where *y_k_* is the output value of node *k*, and *z_k_* is the expected output value.

According to the error back propagation algorithm and the chain rule of differentiation, the update amount ∆*w_jk_* of the weight *w_jk_* is:

(2) $$\Delta w_{jk}=-\eta \cdot \frac{\partial E(w)}{w_{jk}}=\eta \cdot (z_{k}-y_{k})\cdot (1-y_{k}{}^{2})\cdot y_{jk}$$

Among them, *η* is the learning rate, which can be adjusted by the amplitude and pulse width of the voltage pulse.

For hidden nodes, the same can be obtained:

(3) $$\Delta w_{ij}=-\eta \cdot \frac{\partial E(w)}{\partial w_{ij}}=\eta \cdot \sum_{k=1}^{n}\{(z_{k}-y_{k})\cdot (1-y_{k}{}^{2})\cdot w_{jk}\}\cdot (1-y_{j}{}^{2})\cdot y_{ij}$$

It can be seen that as long as the partial derivative of the error to the input of previous layer is deduced from the output layer in turn, the weight update amount of each layer can be obtained.

Figure 3e shows the flow chart of synapse weight training. First, input the binary training image signal into the matrix. The error and weight update amount are calculated according to the output result. The pulse of updating weight in training is the constant square wave of 1.8 V/1 μs. As mentioned earlier, each weight in the network is determined by the difference between the conductance of two memristors. So, each weight has a positive correlation with the resistance of one memristor, and a negative correlation with the resistance of the other memristor. If the weight update amount is positive, a Set pulse is input to the memristor corresponding to the positive value of the weight, and a Reset pulse is input to the memristor corresponding to the negative value. If the weight update amount is negative, input a Reset pulse to the memristor corresponding to the positive value of the weight, and input a Set pulse to the memristor corresponding to the negative value.

To reduce the failure rate of memristors, all memristors in the synaptic weight array are pre-set to low conductance. Figure 3f shows the cumulative distribution of the conductance state of the memristor after removing the failed unit, and the inset shows the conductance distribution diagram. The initial conductance of the device before training was within 5 µS, with an average value of 1.89 µS. It can be seen that in the 1300 memristors, there are 80 failed devices (indicated in blue), accounting for 6.15% of the total.

## 4. Results

### 4.1. Single-Layer Network

**Figure 4:**
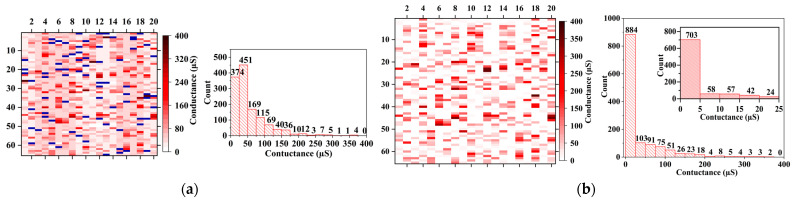
The memristor conductance distribution and the cumulative distribution of the number of devices after 20 trainings, (**a**) before the failed device is replaced, (**b**) after the failed device is replaced. The inset represents an enlarged view of the cumulative number distribution of devices with conductance values ranging from 0 Μs to 25 Μs.

**Figure 5:**
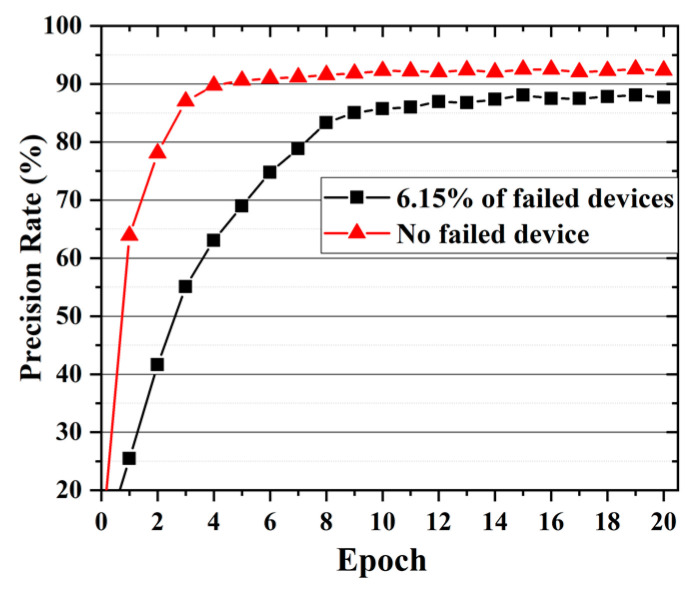
The image recognition accuracy rate of the single-layer network varies with the number of cyclic trainings.

Figure 4a correspond to the conductance of the memristive matrix after 20 cycles of training. It can be seen that, with the exception of failed devices, the conductance of memristor after training ranges from 0 µS to 400 µS. About 63.46% of them is between 0 µS and 50 µS, and the number of devices with conductivity values greater than 50 µS decreases exponentially. According to the curve of recognition accuracy with the number of cycles (Figure 5), it can be seen that the recognition rate of the network gradually increases with the number of cycles training and then tends to be flat. The accuracy of the network after 8 trainings can reach more than 80%. When the number of trainings reaches 12, the recognition rate of the network is basically stable at 87.5%.

As mentioned above, the devices in the system are replaceable. In order to improve the recognition rate of the network, we replace all the failed devices with those that can work normally and train the neural network array again for 20 times. The final conductance distribution of the memristor array is shown in Figure 4b. About 54.08% of the conductance is less than 5 µS. It can be seen that the overall conductance is reduced, and the conductance of more than half of the devices are hardly changes. Figure 5 compares the image recognition accuracy of the network with the number of cyclic trainings in the two cases. It can be seen that failed devices have a greater impact on fewer training times. When there are no failed devices in the networks, the accuracy of image recognition after only 1 training is 63.88%. After 5 training cycles, it finally stabilized at about 92.35%. The recognition accuracy was higher than the 91.71% achieved by Li et al. [26] through a two-layer neural network and 92.13% achieved by Wang et al. [25] through a convolutional neural network.

### 4.2. Double-Layer Network

**Figure 6:**
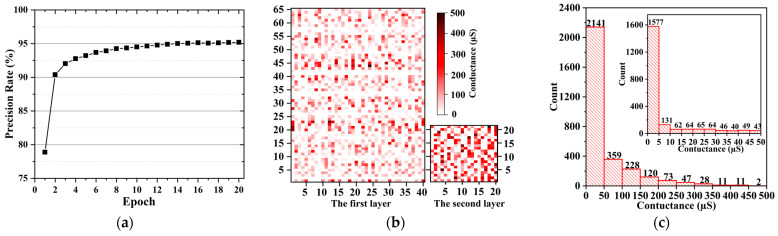
(**a**) The image recognition accuracy rate of the double-layer network varies with the number of cyclic trainings. (**b**) The conductance distribution of the memristor of the double-layer network after training. (**c**) The distribution of the cumulative number of memristor of the double-layer network after training.

In order to further improve the accuracy of network recognition, a double-layer fully connected network containing 20 hidden neurons is constructed. The first and second layers of networks use 65 × 40 and 21 × 20 memristor arrays, respectively. According to Figure 6a, it can be seen that the image recognition accuracy of the two-layer network exceeds 90% after only 2 cycles of training. With the increase of the number of circuit training, the accuracy rate increases slowly and finally reaches about 95.19%.

As shown in Figure 6b, conductance distribution of the memristor array after training is similar to that of a single-layer network, that is, most devices are in a low conductance state. In addition, the overall conductance of the memristor in the second layer network is slightly larger than that of the first layer. Figure 6c shows that about 52.22% of the devices have a conductivity value of less than 5 μS.

**Figure 7:**
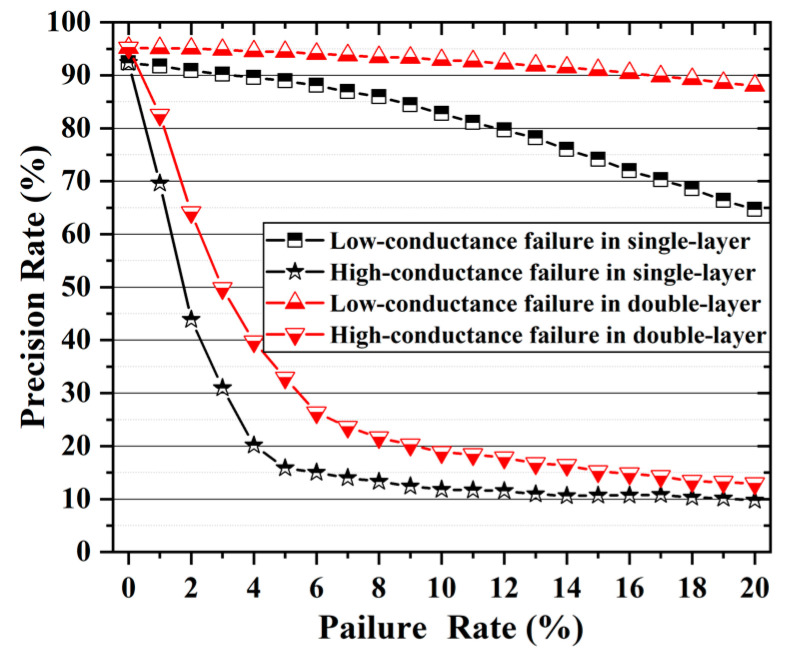
The network image recognition accuracy rate varies with the proportion of failed devices.

In order to study the influence of failed devices on the recognition rate of the network, the memristor of the single-layer and double-layer networks that have been trained are randomly replaced with those in high-conductance failure or low-conductivity failure. Figure 7 indicates the experimentally obtained effect of different proportions of failed devices on the accuracy of network recognition.

It can be seen that devices with low-conductivity failure have little effect on the network recognition rate. When 20% of failed devices appear in the two-layer network (far higher than the actual proportion of failed devices of about 6.15%), there is still a recognition accuracy of 88.06%. The single-layer network has a slightly poorer fault tolerance to low-conductance failed devices, and 20% of failed devices will reduce the recognition accuracy of the network to 64.7%. Regardless of whether it is a single-layer or double-layer network, the recognition accuracy rate drops sharply as the proportion of high-conductance failure devices increases. When the proportion of failed devices exceeds 4%, the recognition accuracy of the single-layer network is just less than 20%. When the proportion of failed devices exceeds 10%, the recognition accuracy of the double-layer network also drops to about 20%. The tolerance for low-conductance failure devices is much greater than that for high-conductance failure devices. This may be explained by the fact that the output current is too high. Excessive current will mask the current signals of other units in the same row in the array, seriously hindering the normal operation of the entire network. Therefore, it is necessary to avoid high-conductance failure devices in the array.

**Figure 8:**
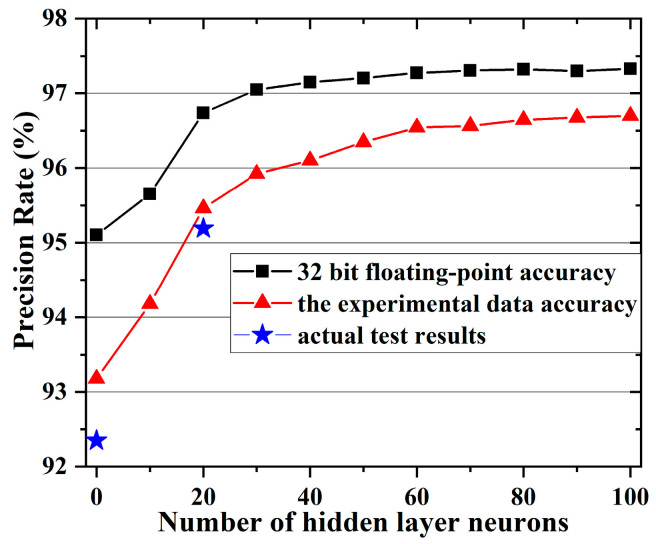
The simulation result of the image recognition accuracy of the double-layer fully connected network changes with the number of neurons in the hidden layer.

The influence of the number of neurons in the hidden layer of the double-layer network on the recognition accuracy is investigated by simulation. Figure 8 shows the simulation results of the image recognition accuracy of the double-layer fully connected network with the number of neurons in the hidden layer. The weight range and adjustment accuracy of the memristor in the simulation are obtained from previous tests (shown in Figure 2c). Taking into account the device-to-device variation, each simulated device is randomly assigned from these 10 sets of data. It compares with the calculation result under 32-bit floating point precision. It can be concluded that as the number of neurons in the middle-hidden layer increases, the accuracy of both increases gradually. The accuracy rate calculated according to the actual adjustment accuracy can reach about 96.57%, which is close to the accuracy rate of 97.33% obtained by the simulation of 32-bit floating point precision. At the same time, it can be seen that the recognition accuracy of the simulated single-layer network and the two-layer neural network containing 20 hidden neurons are 93.18% and 95.46%, respectively, which are similar to the actual test results.

## 5. Conclusions

This paper designs and builds a single-layer and double-layer fully connected network system based on 1T1R array to realize handwritten digital image recognition. For the 1T1R unit, the device can realize a continuous adjustment of the conductivity of about 1 µS~350 µS by applying voltage pulses. By replacing the failed devices, a recognition accuracy rate of 95.19% is obtained in the double-layer neural network. It is found that devices in a low-conductance failure state have little effect on the recognition accuracy of the network, while devices in a high-conductance failure will sharply reduce the recognition accuracy of the network and increase the overall conductance of the memristor. According to the experimental test parameters, the relationship between the image recognition accuracy of the double-layer fully connected network and the number of hidden neurons is achieved by simulation. By increasing the amount of hidden neurons, the recognition accuracy of the network can reach 96.57%. Compared with the simulation results of weight accuracy of the 32-bit floating-point accuracy, the error is below 1%.

## Data Availability

Data are contained within the article.

## Supplementary Materials

The following supporting information can be downloaded at: https://www.mdpi.com/article/10.3390/nano12071075/s1, Figures S1–S4.
