# Supplementary material for: The Image Identification Application with HfO2-Based Replaceable 1T1R Neural Networks
Source: Nanomaterials (Basel). 2022 Mar 25;12(7):1075. doi: 10.3390/nano12071075 (PMC9000711; doi:10.3390/nano12071075)
Supplement: Supplementary file 1 [file nanomaterials-12-01075-s001.zip › nanomaterials-1627600-supplementary.pdf]

Supplementary Materials

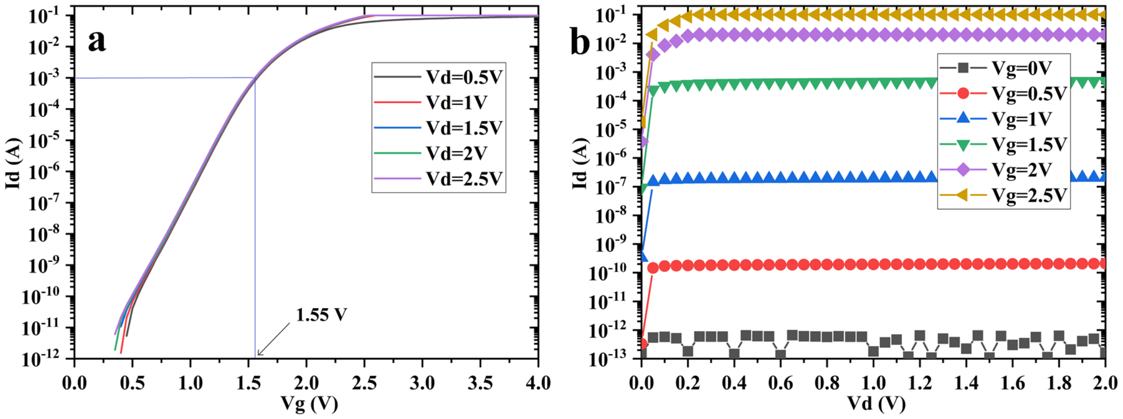

Figure S1. (**a**) The transfer characteristic curve and (**b**) output characteristic curve of MOSFETs. When Vg = 0 V, the drain current is less than 1 pA, and the memristor connected in series with it is considered to be completely turned off. The setting of the current limiting current in the Set process is determined by the gate voltage of the MOS. For example, to set a limit current of 1 mA, according to the transfer characteristics of Figure 1a, it can be known that the gate voltage needs to be set to 1.55 V. In the process of Read and Reset, the gate voltage will be set above 2.5 V, which reduces the voltage dividing effect of transistor on-resistance on the memristor.

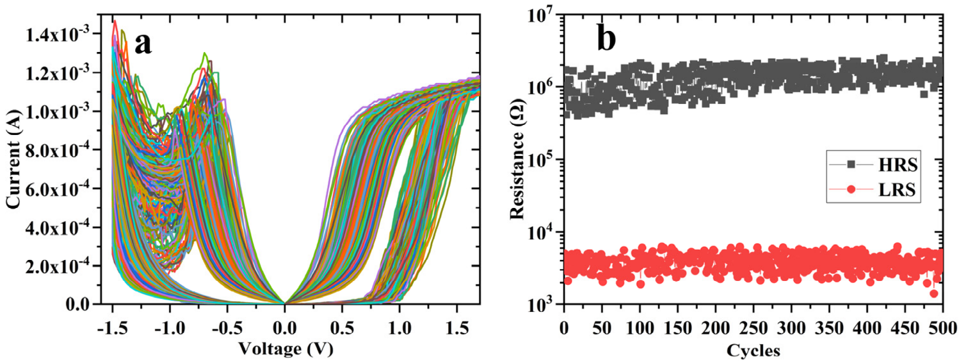

Figure S2. 1T1R unit durability test. (**a**) DC scanning curve of 500 cycles and (**b**) HRS/LRS resistance distribution. The change of current in Set and Reset processes is not abrupt, which means that the device has the potential of multi-value storage. The high/low resistance state resistance distribution of the unit is relatively concentrated, with a switching ratio of about 500, and there is still no degradation after 500 cycles.

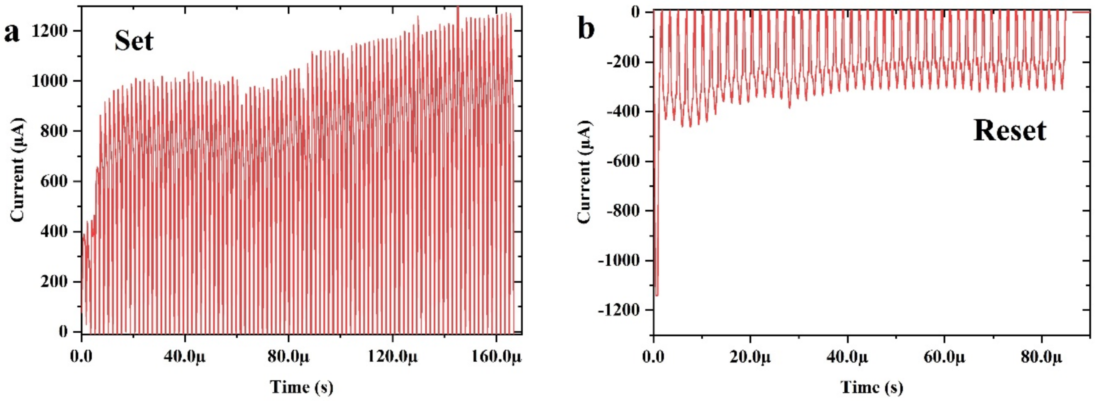

Figure S3. Typical impulse response results of pure HfO_2 memristor. 1.8 V/ 1 μs and −1.8 V/ 1 μs voltage pulses were used in (**a**) the SET process and (**b**) the RESET process, respectively.

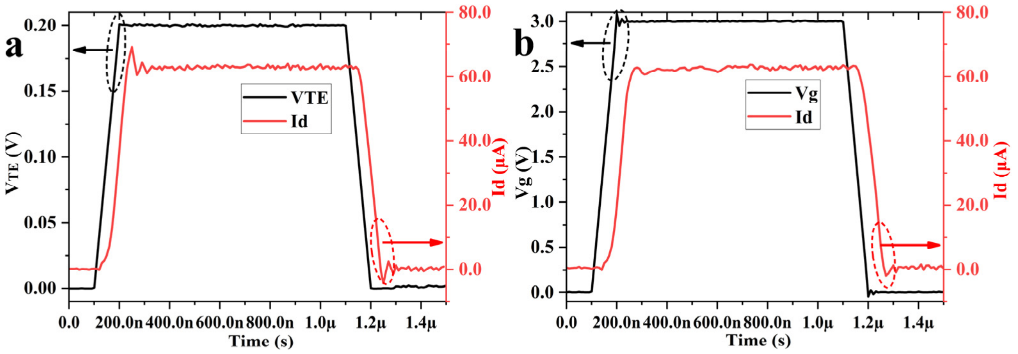

Figure S4. 1T1R unit reading speed test. (**a**) transistor gate voltage Vg=3V, apply voltage pulse V_{TE} to memristor top electrode and (**b**) memristor top electrode V_{TE}=0.2V, apply voltage pulse Vg to transistor gate. The turn-on and turn-off delays of the unit are both less than 100ns, which is mainly limited by the minimum 100ns rising and falling edges of the WGFMU module of the B1500A. There is a longer delay when applying the read voltage to the gate, mainly due to the inherent turn-on and turn-off times of the transistor. It shows that the delay caused by the discrete connection of the device has little effect on the whole.
